# Rhinocerebral Mucormycosis in a COVID-19 Patient from Nepal: A Case Report

**DOI:** 10.31729/jnma.6904

**Published:** 2021-07-31

**Authors:** Sanjay Kumar Gupta, Pallawi Jyotsana, Avilasha Singh, Diwakar Phuyal, Parbej Allam

**Affiliations:** 1 Department of General Practice and Emergency Medicine, Tribhuvan University Teaching Hospital, Maharajgunj, Kathmandu, Nepal; 2 Kathmandu Medical College, Sinamangal, Kathmandu, Nepal; 3 Nepal Medical College and Teaching Hospital, Jorpati, Kathmandu, Nepal; 4 Tribhuvan University Teaching Hospital, Maharajgunj, Kathmandu, Nepal

**Keywords:** *case report*, *COVID-19*, *mucormycosis*, *Nepal*

## Abstract

With the surge of cases during the second wave of COVID-19 in Nepal, a number of mucormycosis coinfection cases have also come to our attention. We present a case of a 65-year-old female who was admitted to our emergency department with complaints of pain, swelling, and tingling sensation of the left side of the face along with blood-tinged nasal discharge for 20 days. She had been tested positive for COVID-19 a month back and managed with oxygen support and corticosteroids. Magnetic Resonance Imaging showed invasive fungal sinusitis, with the positive black turbinate sign and mild extension along with the dura mater of the left temporal lobe, and left cavernous sinus. She was diagnosed with rhinocerebral mucormycosis and managed with systemic antifungal therapy and insulin. As per the treatment modality, surgical debridement could not be done because the patient did not give consent.

## INTRODUCTION

Mucormycosis is an opportunistic infection caused by a group of fungus called mucormycetes. It causes a rapidly progressive vasotrophic infection that can present as rhinocerebral, pulmonary, cutaneous, gastrointestinal, and disseminated. Among which pulmonary and rhinocerebral are the most common presentations.

A wide range of bacterial and fungal infections are being associated with SARS-CoV 2 infection.^1^ Recent surge in the incidence of mucormycosis in COVID-19 patients in countries like India and Nepal has only provided us with more evidence. Saying that it is also crucial to consider other risk factors such as corticosteroid use, comorbidities like diabetes mellitus.^2^

Herein, a case of rhinocerebral mucormycosis treated at a single tertiary care center in Nepal was reviewed.

## CASE REPORT

A 65-year-old female was admitted to the Emergency Department of Tribhuvan University Teaching Hospital (TUTH), on 2078/02/30 with complaints of pain, swelling, and tingling sensation of the left side of the face, along with blood-tinged nasal discharge for 20 days. She was tested positive for COVID-19 one month back in Birgunj Metro City hospital and was a known case of Type 2 Diabetes Mellitus (DM) under medications for eight years. She was admitted there with complaints of fever, cough, and shortness of breath for 2 days and oxygen saturation of 89%. She was managed with oxygen support, Inj Dexamethasone 6mg, Inj Piperacillin and Tazobactam 4.5g, Inj. Levofloxacin 500mg and discharged after ten days.

During admission, the deranged investigations were; Lymphocyte count 20% (25-45%), C-reactive protein 52mg/l (0.068-8.2mg/l), Random Blood Sugar 3.8mmol/L (3.8-7.8), Urea 140uMol/L (40-110), Creatinine 7.2mmol/l (1.6-7.0), Sodium 134mEq/L (135146), Prothrombin time 16sec (10-12), Prothrombin time control 13.5sec (10-12). Histology reports on the KOH mount showed broad hyaline aseptate hyphae with angle branching. Our patient developed compensated respiratory alkalosis with metabolic acidosis.

**Figure 1:**
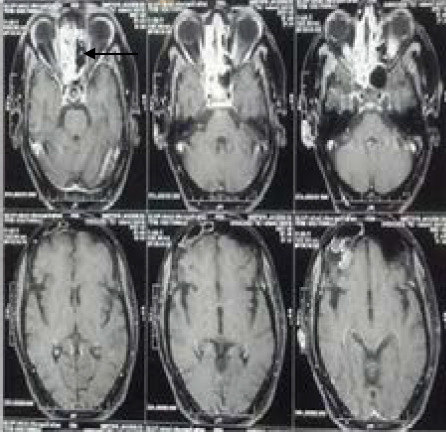
T1 Post-contrast MRI showing nonenhancing tissue in the left ethmoid sinus giving the Black turbinate sign. No intracranial and intraorbital extension noted.

She was consulted by the neurosurgery and Ear, nose, and throat (ENT) departments. Her Glasgow Coma Scale was Eye 4, Verbal 5, Motor 6 pupil bilateral 3mm, motor 5/5, sensory intact. Magnetic Resonance Imaging showed invasive fungal sinusitis, with the positive black turbinate sign and mild extension along the dura of the left temporal lobe, and left cavernous sinus (Figure 1).

She was managed with Inj. Liposomal Amphotericin (B) 300mg and Inj Lispro (Insulin) and Inj. Lantus (Insulin) for four days. She was advised for left endoscopic debridement surgery which was denied by the patient and the patient party. The patient took leave against medical advice.

## DISCUSSION

The Lancet reported that the incidence of Mucormycosis had risen to 14872 cases as of May 28, 2021, in India alone with a total of 90 deaths attributable to mucormycosis.^3^ In a study done by Fouad, et al. from Mar-Sept 2020, 12 cases, with Rhino-orbital cerebral mucormycosis were identified over 6 months (compared to one case in 2017, two in 2018, and one in 2019, all in the corresponding 6-month interval).^4^

Similar to our case a number of reported cases of mucormycosis in COVID-19 patients also had a history of diabetes mellitus. A systematic review by Singh, et al. reported that out of the 101 cases of mucormycosis seen by them 72 cases had preexisting Diabetes Mellitus and 2 new-onset diabetes mellitus were seen.^2^ In another similar study out of the eighteen patients with Diabetes Mellitus and report of blood glucose and acid status, 8 (8/18, 44%) were in diabetic ketoacidosis (DKA) at the time of presentation.^5^ Likewise another case report showed two fatal cases of rhino-orbital-cerebral mucormycosis associated with COVID-19 infection where both patients had pre-existing diabetes mellitus type 2, were treated with corticosteroids and developed ketoacidosis.^6^ In our patient compensated respiratory alkalosis with metabolic acidosis was seen.

In a study done in India among 2826 patients on Rhino-orbital-cerebral mucormycosis, 87% of the patients were treated with corticosteroids, (21% for >10 days).^7^ A similar case was reported by Ahmadikia, et al. where they described systemic corticosteroid as a double-edged sword in COVID-19 patients.^8^ Our patient was also managed with IV dexamethasone of 6mg for 10 days.

A case series done by Ashour, et al. discussed the imaging modalities used for Rinocerebral mucormycosis where fives were diagnosed via contrast-enhanced computed tomography scan whereas contrast-enhanced MRI was used in 3.^9^ In our case we used plain MRI for diagnosis.

The retrospective study done by Kolekar, et al. showed the treatment modality to be control of underlying disease, systemic antifungal therapy, and repeated surgical debridement of necrotic tissues.^10^ But since our patient did not consent surgical debridement could not be done and the patient took leave against medical advice.
